# Mitochondria as Sensors of Amyloid‐Beta Toxicity and Regulators of Inflammation in Cerebrovascular Endothelial Cells

**DOI:** 10.1002/alz.092856

**Published:** 2025-01-03

**Authors:** Rebecca M Parodi‐Rullan, Ashley M Carey, Silvia Fossati

**Affiliations:** ^1^ Lewis Katz School of Medicine, Temple University, Philadelphia, PA USA; ^2^ Alzheimer’s Center at Lewis Katz School of Medicine, Temple University, Philadelphia, PA USA

## Abstract

**Background:**

The blood‐brain barrier (BBB) is a crucial regulator of cerebral homeostasis and function. Cerebrovascular endothelial cells (EC) are important components of the BBB, and EC damage and/or dysfunction may result in defects in brain clearance and perfusion, microhemorrhages, inflammation, and neurodegeneration. In addition to EC damage resulting from the presence of amyloid‐beta (Aβ) in Alzheimer’s Disease (AD) and Cerebral Amyloid Angiopathy (CAA), the presence of cardiovascular risk factors (CVRF) may further exacerbate cerebrovascular function and neurodegeneration. Particularly, ECs may be the most susceptible to Aβ and CVRF damage leading to the initiation and/or perpetuation of an inflammatory cascade in neurodegenerative diseases. Brain ECs are highly dependent on glycolysis and their mitochondria have important roles as signaling organelles, sensing cellular damage and mediating downstream pathways such as inflammation. Here, we aim to understand the mechanistic nature of endothelial mitochondrial dysfunction and its elicited pathways in Aβ and mixed (Aβ+CVRF) vascular pathologies. Understanding the mechanisms by which the mitochondria may mediate EC inflammation could pave the way to new roads for therapeutic advancements in the field.

**Method:**

Human brain microvascular ECs were challenged with Aβ, Homocysteine (Hcy, a CVRF), or the combination. Mitochondrial ultrastructure, metabolic function, and inflammatory cascades were assessed.

**Result:**

The presence of Aβ, but not Hcy, increased mitochondrial ROS production (mtROS) and induced deficits in mitochondrial respiration concomitant with a decrease in mitochondrial ATP production. Deficits in mitochondrial respiration were accompanied by an increase in glycolytic ATP production and, to some extent, the expression of proteins involved in glycolytic regulation. Mitochondrial fragmentation, as an indicator of mitochondrial damage, was assessed ultrastructually and by the analysis of the expression of fission proteins. Markers of EC activation were assessed to link mitochondrial dysfunction to EC inflammation.

**Conclusion:**

Deficits in mitochondrial function and metabolism were associated with Aβ but not Hcy, and accompanied by the release of mtROS, a mitochondria damage associated molecular patterns (mtDAMP). This, in turn, was associated to an increase in EC inflammatory markers and barrier permeability. Overall, our results reveal a novel role for mtDAMPs in vascular dysfunction in the presence of Aβ and/or Hcy.

